# Identifying pathological myopia associated genes with GenePlexus in protein-protein interaction network

**DOI:** 10.3389/fgene.2025.1533567

**Published:** 2025-03-05

**Authors:** Yuanyuan Luo, Yihan Wang, Lin Liu, Feiming Huang, Shiheng Lu, Yan Yan

**Affiliations:** ^1^ Department of Ophthalmology, Renji Hospital, School of Medicine, Shanghai Jiao Tong University, Shanghai, China; ^2^ School of Life Sciences, Shanghai University, Shanghai, China; ^3^ Department of Ophthalmology, Shanghai Eye Diseases Prevention and Treatment Center/Shanghai Eye Hospital, School of Medicine, Tongji University, Shanghai, China; ^4^ Department of Ophthalmology, Eye and ENT Hospital, Fudan University, Shanghai, China

**Keywords:** pathological myopia, high myopia, degenerative myopia, disease gene, GenePlexus, DisGeNET

## Abstract

**Introduction:**

Pathological myopia, a severe form of myopia, is characterized by an extreme elongation of the eyeball, leading to various vision-threatening complications. It is broadly classified into two primary types: high myopia, which primarily involves an excessive axial length of the eye with potential for reversible vision loss, and degenerative myopia, associated with progressive and irreversible retinal damage.

**Methods:**

Leveraging data from DisGeNET, reporting 184 genes linked to high myopia and 39 genes associated with degenerative myopia, we employed the GenePlexus methodology in conjunction with screening tests to further explore the genetic landscape of pathological myopia.

**Results and discussion:**

Our comprehensive analysis resulted in the discovery of 21 new genes associated with degenerative myopia and 133 genes linked to high myopia with significant confidence. Among these findings, genes such as ADCY4, a regulator of the cAMP pathway, were functionally linked to high myopia, while THBS1, involved in collagen degradation, was closely associated with the pathophysiology of degenerative myopia. These previously unreported genes play crucial roles in the underlying mechanisms of pathological myopia, thereby emphasizing the complexity and multifactorial nature of this condition. The importance of our study resides in the uncovering of new genetic associations with pathological myopia, the provision of potential biomarkers for early screening, and the identification of therapeutic targets.

## 1 Introduction

Myopia is a pathological condition that affects distance vision (Ohno-Matsui et al., 2016; Foster and Jiang, 2014). As a common disease, the eye focus disorder can be corrected with eyeglasses, contact lenses or surgery. According to International Myopia Institute (IMI) white papers (Resnikoff et al., 2019; Tahhan et al., 2023), more than 30% of the world population is suffering from myopia, amounting to more than 2 billion people. Specifically, a higher prevalence have been observed in some regions of the world like U.S. (42%), China mainland (47%), and Hong Kong (62%) (Pan et al., 2012; Holden et al., 2016), indicating myopia as an ongoing global health issues. According to predictions, by 2050, more than 40% of people all over the world will suffer from myopia (Holden et al., 2016). Therefore, studying the pathogenesis of myopia constitutes one of the most important parts of current biomedical research.

Although myopia is a common condition characterized by symptoms such as difficulty seeing distant objects, squinting, and eye strain, heterogeneity in myopia pathogenesis persists (Naiglin et al., 1999; Jones and Luensmann, 2012). High myopia and degenerative myopia represent two major classes of myopia, exhibiting similar nearsighted symptoms but differing in their pathological mechanisms (Russo et al., 2014). High myopia is characterized by a group of myopia primarily caused by inherited genetic conditions, resulting in excessive eyeball elongation (Grzybowski and Kanclerz, 2019; Verkicharla et al., 2015), whereas degenerative myopia presents slightly different characteristics. The pathogenesis of degenerative myopia generally involves the damage to the retina (Park et al., 2013; Grossniklaus and Green, 1992; Lam et al., 2005), crucial for capturing vision and transmitting it to the brain. Despite shared clinical features (e.g., blurred vision, eye strain), their divergent pathological trajectories—mechanical stretching in high myopia versus retinal cell death in degenerative myopia—demand subtype-specific diagnostic and therapeutic strategies. Critically, early differentiation remains challenging due to overlapping symptoms, highlighting the need for molecular biomarkers to guide intervention.

Previously, studies on myopia have identified a series of biomarkers for high myopia and degenerative myopia, respectively. For instance, in 2001, Young and King identified several regions within the MYP2 gene on chromosome 18p11.31 associated with autosomal dominant high myopia (Young et al., 2001). In 2004, two genes, MYP7 and PAX6, were identified through a genome-wide scan as being associated with high myopia (Hammond et al., 2004), highlighting the genetic influence on high myopia pathogenesis. Regarding degenerative myopia, inherited retinal degenerations are prevalent in older populations, representing one of the most common causes of blindness in Europe and North America (Francis, 2006; Duncan et al., 2018). Genes like MYP5 (Paluru et al., 2003) and COLA1A (Madhuri et al., 2021) have been identified as pathogenic in retinal degeneration, playing a significant role in the initiation and progression of myopia. However, existing gene catalogs remain incomplete: DisGeNET curates 184 high myopia-associated genes versus only 39 for degenerative myopia, reflecting a critical knowledge gap in the latter’s molecular drivers. Furthermore, conventional genome-wide association studies (GWAS) and linkage analyses face limitations in resolving polygenic interactions and tissue-specific networks, necessitating advanced computational approaches.

Recent efforts to bridge this gap have employed network-based algorithms. For instance, Zhang et al. (2024) utilized the random walk with restart (RWR) method to prioritize candidate genes for both subtypes. While RWR leverages protein-protein interaction (PPI) networks, its reliance on predefined seed genes and static network topology may overlook context-dependent gene functions. Additionally, the lack of integration with functional validation data limits its predictive specificity. These shortcomings emphasize the need for methodologies that synergize multi-omic data, dynamic network modeling, and machine learning to improve biomarker discovery.

To address these challenges, we applied GenePlexus, a novel computational framework that integrates network propagation, functional enrichment, and cross-validation, in conjunction with our screening tests to predict subtype-specific biomarkers of myopia. By analyzing DisGeNET-curated genes (184 for high myopia, 39 for degenerative myopia), our study achieves two advances: (Ohno-Matsui et al., 2016): identifying 21 novel degenerative myopia-associated genes (e.g., THBS1, implicated in collagen degradation and retinal integrity) and 133 high-confidence high myopia-associated genes (e.g., ADCY4, modulating cAMP signaling in axial growth); (Foster and Jiang, 2014); validating the functional relevance of predicted genes through pathway analysis. Our findings not only expand the genetic landscape of pathological myopia but also establish a paradigm for computationally deconvoluting complex ophthalmic disorders.

## 2 Methods

### 2.1 Pathological myopia associated genes

Pathological myopia, a form of myopia characterized by severe nearsightedness and structural changes in the eye, can lead to additional visual impairment. We employed DisGeNET (v7.0, https://disgenet.com/) (Piñero et al., 2019), a comprehensive database aggregating gene-disease associations from diverse sources such as expert-curated databases, scientific literature, and electronic health records, thereby facilitating in-depth genetic research, to collect genes related to pathological myopia. Using the keywords “high myopia” and “degenerative myopia,” we identified genes on two distinct levels. Utilizing DisGeNET, we identified 184 genes associated with high myopia and 39 genes linked to degenerative myopia. The identified 184 high myopia and 39 degenerative myopia genes were subsequently matched to the protein-protein interaction (PPI) network, yielding 170 proteins for high myopia and 34 proteins for degenerative myopia, detailed in Supplementary Table S1.

### 2.2 PPI network

In this research, we focused on identifying proteins associated with high myopia and degenerative myopia, employing GenePlexus within the framework of the PPI network. Serving as the foundation for our exploration, the PPI network, a critical asset for pinpointing and forecasting essential genes, allowed us to utilize its comprehensive structure effectively. In the PPI network, the associations between proteins are clearly displayed. Each protein can be considered by taking all other proteins as background. The method based on the PPI network can systematically overview all proteins and investigate individual protein at a system level. This is the special merit compared with the traditional methods that can only investigate proteins individually. Here, we revealed new candidate genes associated with high myopia and degenerative myopia, thereby broadening the scope of knowledge beyond existing genetic findings. This PPI network has wide applications in tackling various protein-related problems (Pan et al., 2022; Zhang and Chen, 2020; Zhao et al., 2020; Zhao et al., 2019; Huang et al., 2023; Li et al., 2022a; Huang et al., 2024).

The data for our study were sourced from the STRING database (version 10, https://www.string-db.org/) (Szklarczyk et al., 2023), comprising 4,274,001 interactions among 19,247 human proteins, as detailed in the file “9606. protein.links.v10. txt.gz.” These interactions are depicted as links between pairs of proteins, each identified by their Ensembl IDs. Furthermore, the PPIs are assigned a confidence score ranging from 1 to 999. This scoring system derives from the amalgamation of various sub-scores, including measures of neighborhood, fusion, co-occurrence, co-expression, experimental evidence, database integration, and text mining analysis. These metrics provide a multifaceted evaluation of the proteins’ interactions, accounting for factors such as genomic proximity, gene fusion events, occurrences across species, co-expression patterns, and corroboration from scientific literature.

For the construction of the PPI network, 19,247 human proteins were utilized as nodes. Connections (or edges) between these nodes were established based on the existence of a PPI between the corresponding proteins, given that they had a confidence score above zero. The strength of these interactions is quantitatively represented in the edge weights, with higher scores indicating stronger associations. This methodological approach enabled a detailed and nuanced assessment of the protein interactions, facilitating the identification of potential genetic markers for high and degenerative myopia with greater precision.

### 2.3 GenePlexus

Addressing the challenge of identifying genes within a molecular network G that are closely associated with a specific set of genes S , linked to certain functions, diseases, or traits, Liu et al. introduced GenePlexus (Liu et al., 2020a). This tool is adept at assessing the relevance of genes within network G to the set S . GenePlexus characterizes the gene network G through three distinct types of matrices, termed representation matrices: the adjacency matrix, the influence matrix, and the node embedding matrix. These matrices utilize network neighborhoods as features, with each row signifying a gene and each column a feature. For a given gene set S , genes within this set are treated as positive samples. By omitting these positives and genes from sets similar to S , the remaining genes in comprehensive gene set collections serve as negative samples. These samples, encoded via a chosen representation matrix, facilitate the training of a logistic regression model with L2 regularization. This model quantifies the relationship between each gene in G and the set S . In 2023, Mancuso et al. developed PyGenePlexus (Mancuso et al., 2023), a Python package implementing GenePlexus. With PyGenePlexus, users can input a gene set S , select a gene network G , and opt for a representation matrix type, obtaining outputs that include the relevance of each gene in G to S (measured by a probability), connections of top relevant genes, closely related gene sets (encompassing known pathways, processes, and diseases), and prediction accuracy (evaluated via cross-validation). Through extensive comparison across numerous prediction tasks and multiple gene networks, the authors demonstrated that GenePlexus surpasses traditional label propagation methods, underscoring its value to the research community (Liu et al., 2020a).

### 2.4 Screening tests

Our results suggest that certain candidate genes, as identified by GenePlexus, exhibit a strong association with the structure of the PPI network and may display distinct behaviors. For instance, certain nodes might have a higher likelihood of being identified as key nodes, irrespective of the chosen seed nodes. Conversely, candidate genes exhibiting more substantial connections with previously validated genes are more likely to be identified as novel genes associated with high myopia and degenerative myopia. To further refine the identification process for these critical candidate genes, we designed three additional screening tests.

#### 2.4.1 Permutation test

The architecture of the PPI network plays a pivotal role in determining the results generated by GenePlexus, with specific genes within the network being inherently more likely to be flagged due to their positions. However, it's important to note that not all these prominently flagged genes are directly linked to high myopia and degenerative myopia. To effectively sift through these genes, a permutation test was implemented. This began with the creation of one thousand random gene sets, each tagged from $S_{1}$ to $S_{1000}$ , containing a quantity of Ensembl gene identifiers equivalent to the genes associated with high myopia and degenerative myopia. For every one of these sets, the corresponding Ensembl IDs served as initial nodes for analysis in GenePlexus, assigning a unique probability to each gene. A p-value was then calculated for each gene under scrutiny, based on Equation 1:

$$p_{value\left(g\right)}=\frac{N}{1000}$$ (1)

where N denotes the number of random sets. A gene showing a particularly low p-value was thereby considered highly likely to represent a novel pathological myopia associated gene, attributed to its rare occurrence in the random sets. We adopted a p-value cutoff of 0.05, a widely accepted standard for minimizing false positives in statistical analysis. Consequently, genes with p-values under this threshold were classified as candidate genes for further investigation.

#### 2.4.2 Interaction test

This examination focuses on assessing the potential connection between selected genes and severe vision conditions, namely high myopia and its degenerative counterpart. The analysis entails determining how each gene under consideration relates to others that have already been associated with these myopic conditions (Cai et al., 2017; Liang et al., 2021). The intensity of protein interactions, a crucial factor in this assessment, is quantified using confidence scores available in the STRING database. For any given gene, referred to as g , the process involves calculating the maximum interaction score (MIS) it has with any protein $g^{\prime }$ previously linked to pathological myopia, using Equation 2:

$$MIS\left(g\right)=\mathrm{Max}\left\{\begin{array}{l} Q\left(g,g^{\prime }\right):g^{\prime }\;is\;a\;validated\;pathological\;\\ myopia\;associated\;gene \end{array}\right\}$$ (2)

Candidate genes that exhibit high MIS values are considered more likely to be involved in high myopia and degenerative myopia due to the strength of their protein-protein interactions. To narrow down the most promising candidates, we established a minimum MIS value of 0.9, a benchmark reflecting the highest level of confidence according to the STRING database’s scoring system. This stringent criterion ensures that only genes with the most substantial evidence of a link to myopia are selected for further investigation.

#### 2.4.3 Enrichment test

The purpose of this concluding analysis is to enhance the process of selecting candidate genes by examining how closely their functional terms align with those associated with severe myopic conditions, both high and degenerative. A gene’s candidacy is strengthened if its functional descriptors closely resemble those of genes already confirmed to be involved with these types of myopia (Chen et al., 2019; Chen et al., 2016). This resemblance is quantitatively assessed through the calculation of an enrichment score (ES), which is derived using Equation 3:

$$ES\left(g,F\right)=-\log_{10}\left(\sum_{k=m}^{n}\frac{\left(\begin{array}{l} M\\ k \end{array}\right)\left(\begin{array}{l} N-M\\ n-k \end{array}\right)}{\left(\begin{array}{l} N\\ n \end{array}\right)}\right)$$ (3)

Here, F represents a specific GO term or KEGG pathway, N is the total count of human genes, and M is the number of genes annotated by F . Moreover, n indicates the count of genes interacting with gene g as documented in STRING, and m is the number of these interacting genes also annotated by F . For each gene g , a vector $V\left(g\right)$ incorporating enrichment scores across all relevant GO terms and KEGG pathways is created. The relationship between any two genes, g and $g^{\prime }$ , is then assessed by computing the cosine similarity between their vectors, formulated by Equation 4:

$$\Phi \left(g,g^{\prime }\right)=\frac{\;V\left(g\right)\cdot V\left(g^{\prime }\right)}{\left\|V\left(g\right)\right\|\cdot \left\|V\left(g^{\prime }\right)\right\|}$$ (4)

Additionally, for every candidate gene g , the maximum enrichment score (MES) is determined by Equation 5:

$$MES\left(g\right)=\mathrm{Max}\left\{\begin{array}{c} \Phi \left(g,g^{\prime }\right):g^{\prime }\;is\;a\;validated\;pathological\;\\ myopia\;associated\;gene \end{array}\right\}$$ (5)

To single out the most promising candidate genes for a link to high and degenerative myopia, an MES threshold of 0.95 has been established. This threshold serves as a benchmark to identify genes whose functional profiles not only share a high degree of similarity with those known to affect myopia but also suggests a substantial likelihood of their involvement in these conditions.

### 2.5 Outline of the method

**FIGURE 1:**
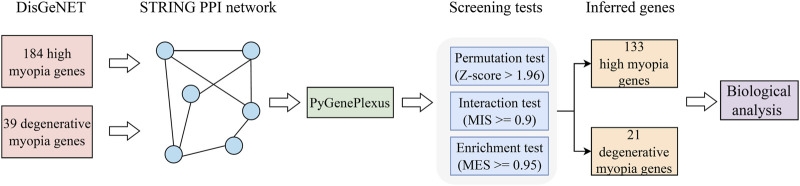
Flowchart depicting the procedure of extracting genes linked to pathological myopia. Initially, we gathered validated genes associated with high myopia and degenerative myopia from the DisGeNET database. Following this, we constructed a PPI network using data from the STRING database, a crucial step in visualizing the complex interplay between proteins. The GenePlexus tool was then utilized on this gene network to highlight candidates with a high likelihood of relevance. Subsequent refinement of these potential candidates was conducted through a series of three distinct screening tests, aiming to distill the list to a final set of genes deemed most pertinent. The culmination of our process involved a detailed evaluation of the relationship between these inferred genes and pathological myopia, employing a literature-based analysis to substantiate their relevance to the disease.

In this study, a computational method was designed to identify latent genes related to high and degenerative myopia. The entire procedures are illustrated in Figure 1. The method started with validated genes of high and degenerative myopia retrieved from DisGeNET. Then, they were fed into GenePlexus to identify possible genes in a PPI network. Finally, these possible genes were filtered by three screening tests (permutation, interaction, and enrichment tests) with specific measurements and thresholds. The identified genes were confirmed by biological analysis.

## 3 Results

### 3.1 Results of the GenePlexus

In this study, we explored the PPI network with the help of GenePlexus, focusing on proteins associated with pathological myopia. These proteins were designated as initial points of interest, or “seed nodes”. For each node within the network, we determined a specific likelihood of association with these seed nodes. Excluding the seed nodes themselves, the likelihoods assigned to all other nodes have been compiled in Supplementary Tables S2, S3. Subsequently, we identified the proteins corresponding to these designated nodes.

To assess the significance of each protein, we performed a permutation test to calculate p-values, with results detailed in Supplementary Tables S2, S3. Initially, proteins with a p-value below 0.05 were considered significant, leading to the identification of 2,600 proteins associated with high myopia and 1,427 proteins related to degenerative myopia. Further analysis was conducted through an interaction test, which assigned a MIS to each protein, as outlined in Supplementary Tables S2, S3. Proteins with an MIS of 0.9 or higher were selected, totaling 855 proteins for high myopia and 144 for degenerative myopia. To further evaluate their significance, we conducted an enrichment test on these filtered proteins. This test calculated the MES for each protein, with a threshold set at 0.95. Proteins below this cutoff were excluded, resulting in a final list of 133 proteins for high myopia and 21 for degenerative myopia, detailed in Supplementary Tables S2, S3. These proteins, termed “inferred proteins” in subsequent analyses, are considered to substantially correlate with the conditions of high myopia and degenerative myopia.

### 3.2 Associations between inferred proteins and validated proteins

**FIGURE 2:**
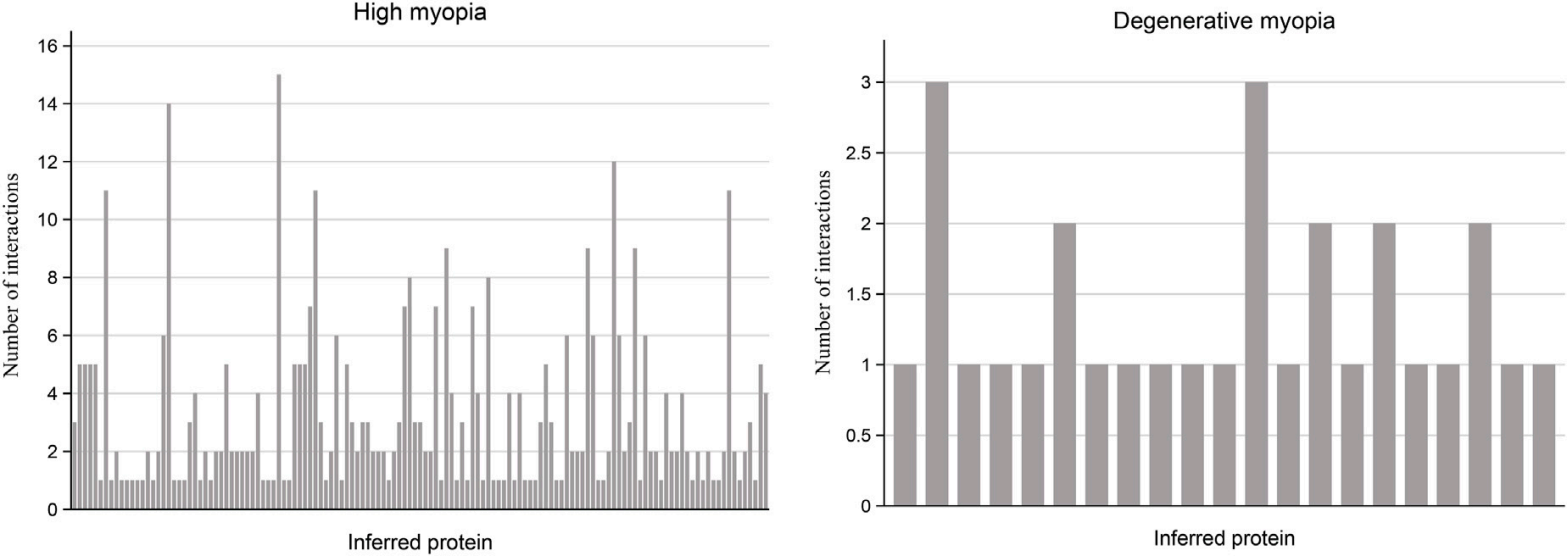
Bar plot illustrating the frequency of interactions between predicted proteins and known proteins linked to high myopia and degenerative myopia with high confidence scores. Among the recently discovered genes associated with high myopia and degenerative myopia, almost all have at least one interaction with known disease-related genes in the PPI network, indicating a strong connection between these inferred proteins and pathological myopia.

In our research, we undertook multiple analyses to verify the reliability of proteins forecasted to be relevant to high and degenerative myopia. Specifically, we measured the number of interactions each predicted protein had within the PPI network with genes linked to these myopic conditions, setting a threshold for confidence scores at 0.9. The outcomes of these measurements are visually summarized in a bar plot illustrated in Figure 2.

For the proteins associated with high myopia, our analysis revealed that 133 of them are connected to at least one gene within the PPI network known to be associated with high myopia. Notably, the protein HN1, identified by the code ENSP00000346839, was distinguished by its linkage to 15 genes related to high myopia, suggesting a potentially significant relationship between HN1 and this condition. Conversely, regarding degenerative myopia, 21 predicted proteins were identified as having at least one connection to a gene associated with degenerative myopia within the PPI network. By examining and comparing the connections between genes inferred from our study concerning high and degenerative myopia, and their specific disease-related genes, we ascertained that the genes identified through our computational analysis are strongly connected to both conditions. This linkage indicates a probable role of these inferred genes in the development and progression of high and degenerative myopia, thereby underscoring the accuracy of our research methodology.

## 4 Discussion

**FIGURE 3:**
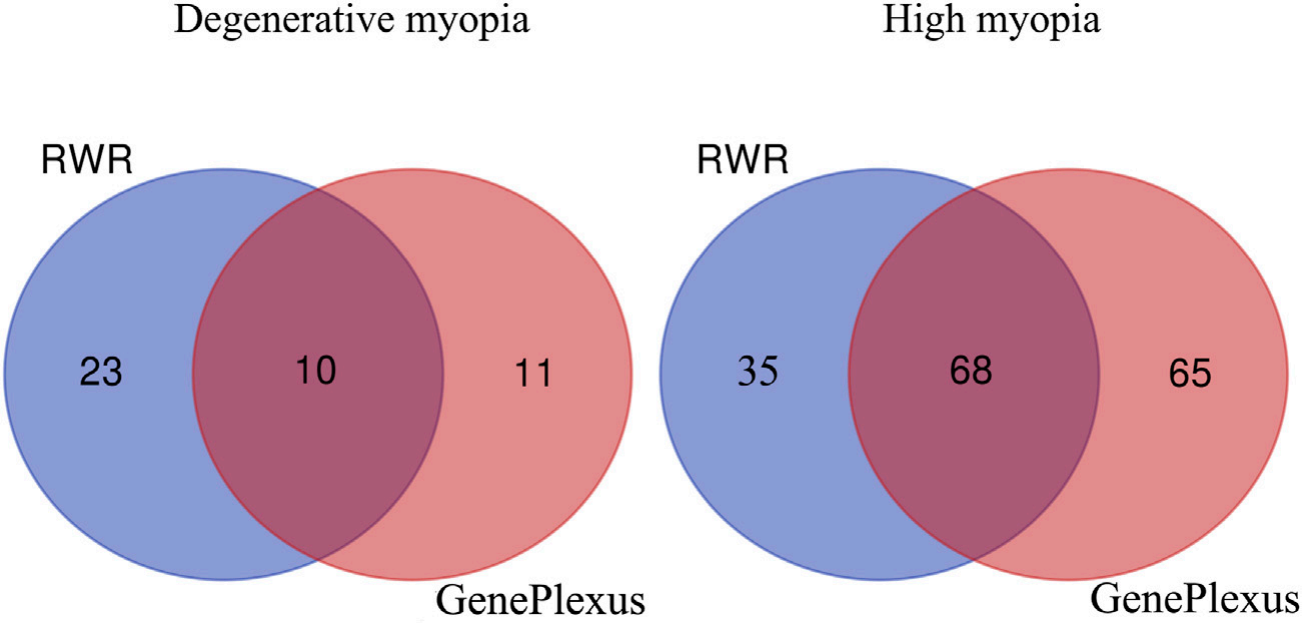
Venn plot of pathological myopia-related genes identified based on the RWR method and those identified based on GenePlexus.

In this study, we introduced GenePlexus to predict novel genes associated with high myopia and degenerative myopia, building upon previously reported biomarkers for these two subtypes of myopia. We identified a series of novel biomarkers and compared our findings with the previous study using RWR algorithm (Zhang et al., 2024). This comparison revealed some common genes between the two methods, as illustrated in Figure 3. The detailed genes in three parts of each Venn diagram are listed in Supplementary Table S4. A literature review confirmed that some predicted myopia biomarkers identified in our study are associated with related pathological mechanisms, thereby validating the efficacy and accuracy of our predictions. The genes exclusively identified in this study are listed in Table 1.

**TABLE 1:** Latent biomarkers identified in this study.

Ensembl ID	Gene symbol	Description	Degenerative myopia	High myopia
ENSP00000238682	TGFB3	Transforming growth factor beta 3	√	✕
ENSP00000260356	THBS1	Thrombospondin 1	√	✕
ENSP00000355645	ACTA1	Actin alpha 1, Skeletal muscle	√	✕
ENSP00000267843	FGF7	Fibroblast growth factor 7	✕	√
ENSP00000312126	ADCY4	Adenylate cyclase 4	✕	√
ENSP00000354558	MTOR	Mechanistic target of rapamycin kinase	✕	√
ENSP00000363708	BMPR2	Bone morphogenetic protein receptor type 2	✕	√
ENSP00000341551	SMAD4	SMAD family member 4	✕	√

### 4.1 Validated biomarkers for degenerative myopia

Comparing our findings with previous predictions based on RWR method, we identified 10 shared genes associated with degenerative myopia predicted by both methods. Notably, *SMAD5* (ENSP00000441954) emerged as the first in the prediction list. *SMAD5* is a crucial component of the transforming growth factor beta (TGF-beta) signaling pathway (Gerjevic et al., 2012; Liu et al., 2013). According to recent publications, in 2017, Fisichella has summarized the specific role of TGF-beta signaling pathway for degeneration associated eye diseases including myopia (Fisichella, 2017), validating our prediction. Among the genes we predicted, *TGFBR1* (ENSP00000364133) is notable for encoding a protein that interacts with type II TGF-beta receptors (Vander et al., 2018). Similarly, considering the specific role for TGF-beta signaling during retinal degeneration (Wang et al., 2019; Saika et al., 2009; Ma et al., 2019), it is reasonable for us to also predict such gene as degenerative myopia biomarkers, validating the efficacy of RWR and GenePlexus methods for degenerative myopia genes prediction. Additionally, *WNT7A* (ENSP00000285018), identified by both methods, is recognized for its involvement in developmental and homeostasis pathways (Lan et al., 2019). During the pathogenesis of degenerative myopia, *WNT7A* has been shown to regulate the plasticity of human retinal pigment epithelial cells, functionally associated with degenerative myopia (Kuznetsova et al., 2016). WNT7A-PAX6 signaling pathway has also been validated to be essential for retinal degeneration, associated with disease animal model design for degenerative myopia (Liu et al., 2017), implying the specific role of such gene during myopia pathogenesis. *CDH1* (ENSP00000261769) is also one of the most important gene associated with degenerative myopia and has been selected by both methods. According to recent publications, retinal cadherins, encoded by *CDH1 in situ*, has been shown to be functionally associated with degenerative change in eye retina tissues (Yusuf et al., 2022). Cadherin associated pathways have also been shown to be pathological during the initiation and progression of myopia (Yang et al., 2022), consistent with our prediction on the specific role of CDH1 using both methods.

### 4.2 Biomarkers for degenerative myopia only predicted by GenePlexus

In addition to the gene identified by both methodologies as previously discussed, the GenePlexus approach has also pinpointed novel biomarkers linked to myopia. Specifically, it recognized two genes within the TGF-Beta signaling pathway—*TGFB3* (ENSP00000238682) and *TGFB2* (ENSP00000355896)—as being associated with degenerative myopia, a connection not made by the RWR method. Given the crucial role of the TGF-beta signaling in the pathogenesis of degenerative myopia, identifying these genes as potential biomarkers is well-founded (Fisichella, 2017). As two components of the signaling pathway, it is reasonable to predict such two genes as potential biomarkers. *THBS1* (ENSP00000260356) is a subunit of a disulfide-linked homotrimer protein, functionally associated with cell-cell/cell-matrix interactions (Liu et al., 2020b; Duan et al., 2021). During the pathogenesis of degenerative myopia, increased collagen degradation associated matrix remodeling in retina has been shown to induce degenerative myopia (Ouyang et al., 2019; Pugazhendhi et al., 2020). Considering the specific role of *THBS1* for collagen degradation (Wen et al., 2023; Inoue et al., 2013), its specific contribution on the pathogenesis of myopia can be validated. *ACTA1* (ENSP00000355645) is another specific gene predicted to be associated with degenerative myopia. *ACTA1* has been reported to participate in retinal development in guinea pig models (Srinivasalu et al., 2020). Degenerative change of extracellular matrix has been shown to be functionally associated with abnormal expression level of ACTA during myopia pathogenesis (Srinivasalu et al., 2020), validating the predictive efficacy of our newly presented GenePlexus based model. Novel predicted genes have also been shown to be functionally supported by recent publications to be associated with degenerative myopia, implying that GenePlexus is also an effective tool to identify disease subtyping associated biomarkers.

### 4.3 Validated biomarkers for high myopia

Using GenePlexus, we validated a set of biomarkers for high myopia previously predicted by the RWR method. For example, *COL1A2* (ENSP00000297268), a component of type I collagen, has been identified as playing a significant role in extracellular matrix remodeling (Lamandé and Bateman, 2020). A 2021 study by researchers at Shanghai Ninth People’s Hospital, China, confirmed the link between type I collagen and high myopia mediated by microRNA-29a, supporting our findings (Zhu et al., 2022). Another gene associated with high myopia is *SOX9* (ENSP00000245479), crucial for chondrocyte differentiation and skeletal growth (De Crombrugghe et al., 2000; Akiyama et al., 2002). Its interaction with BMP-2 and its role in scleral remodeling have been linked to high myopia (Li et al., 2015), validating our prediction. In 2012, researchers from Johnson and Johnson Vision Care also recognized specific role of *SOX9* for extreme myopia (Ritchey et al., 2012). Additionally, *GUCY2D* (ENSP00000254854), which regulates cyclic GMP synthesis (Wimberg et al., 2018; Xue et al., 2019), has variants associated with high myopia, as demonstrated through genetic analyses (Haarman et al., 2022). Different direct protein functional associations, associations between *GUCY2D* and high myopia have been widely reported with a series of genetic association analyses (Haarman et al., 2022; Lazar et al., 2015; Stunkel et al., 2018). Local genetic variants around *GUCY2D* contributes to the pathogenesis of high myopia (Haarman et al., 2022; Koenekoop et al., 2007), which further indicates potential relationships between our predicted gene and such disease. *SPP1* (ENSP00000378517) encodes a non-collagenous bone protein participating in cell-matrix interaction (Kramerova et al., 2019; Erikson et al., 2010). During the pathogenesis of high myopia, *SPP1* has been shown to participate in the abnormal regulation of retinal pigment epithelium metabolism and further contributes to the pathogenesis of high myopia (Zhang, 2013). Familial genomic studies have pointed to *SPP1* as a candidate gene for high myopia (Sánchez-Cazorla et al., 2023). These findings, validated by recent research, confirm the effectiveness and accuracy of the GenePlexus and RWR approaches in identifying relevant genes for high myopia.

### 4.4 Biomarkers for high myopia only predicted by GenePlexus

Comparing with RWR predicted high myopia associated genes, we further predicted variousGenePlexus specific high myopia associated genes, which have also been validated by recent publications, implying the efficacy and accuracy of GenePlexus approach. The first predicted gene is *FGF7* (ENSP00000267843), a functional fibroblast growth factor regulating cell growth, tissue repair and morphogenesis (Nunes et al., 2016; Farooq et al., 2021; Prudovsky, 2021). Associated with *FGF10*, *FGF7* has been confirmed to regulate specific mesenchymal-epithelial transition pathologically associated with extreme high myopia (Prudovsky, 2021), validating our prediction. The next predicted gene is *ADCY4* (ENSP00000312126). *ADCY4* is also a regulator for the secondary messenger cyclic adenosine monophosphate (cAMP) (Fan et al., 2019). The level of secondary messenger cyclic adenosine monophosphate in retina has been shown to be associated with the pathogenesis of high myopia by interfering with collagen metabolism (Zhou et al., 2012; Chun et al., 2015; Zhao et al., 2021). Therefore, it’s reasonable to speculate that *ADCY4*, as cAMP regulator is functionally associated with high myopia. *MTOR* (ENSP00000354558) encodes a serine/threonine protein kinase regulating cell metabolism, growth, and survival (Bhalla et al., 2017; Wullschleger et al., 2006). Gene polymorphisms of *MTOR* has been shown to be highly connected with myopia severity (Li et al., 2022b). *BMPR2* (ENSP00000363708) is also a member of serine/threonine kinases participating in adipogenesis (Motomura et al., 2019; Schleinitz et al., 2011). During the pathogenesis of high myopia, *BMPR2* has been shown to be associated with bidirectional regulator effects on retinal pigment epithelium (Zhang et al., 2012). Therefore, it is reasonable to predict such gene as a potential biomarker for high myopia. *SMAD4* (ENSP00000341551) is a member of Smad family functionally associated with TGF-beta signaling (Zhao et al., 2018; Liu et al., 2023). In 2021, researchers from Fudan University confirmed aberrant TGF-beta signaling and SMAD signaling activation during the pathogenesis of high myopia (Zhu et al., 2021), validating our prediction. As we have discussed above, GenePlexus also recognized a series of novel high myopia biomarkers validated by previous publications, implying the efficacy and accuracy of such computational methods comparing with previous RWR methods.

In conclusion, as highlighted previously, the biomarkers we predicted for distinguishing between high myopia and degenerative myopia have been corroborated by recent scholarly articles. A comparison with the RWR method revealed that approximately 50% of our predicted biomarkers were also identified by RWR, underscoring the effectiveness and precision of predictions made using GenePlexus. This demonstrates that the integration of machine learning models like GenePlexus into the research process can successfully unveil novel biomarkers for various myopia subtypes. Furthermore, employing a range of effective machine learning models enhances the overall accuracy and efficiency of predictive modeling and biomarker discovery, suggesting a robust approach for the identification of disease-specific markers.

### 4.5 Enrichment analysis

**FIGURE 4:**
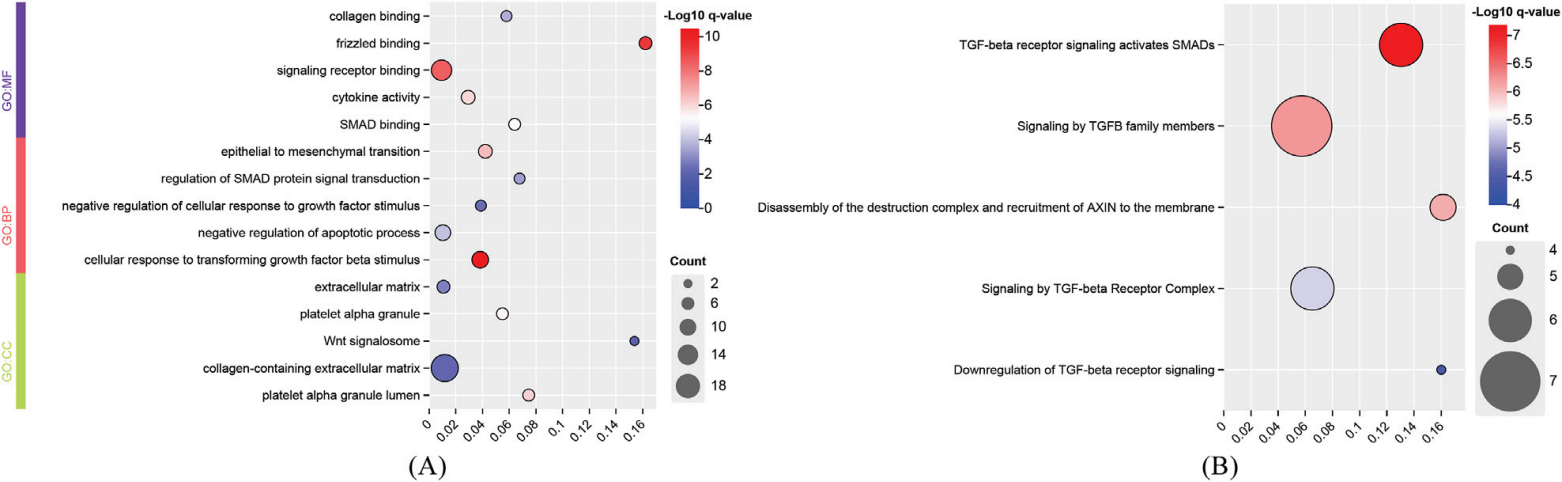
Enrichment analysis results of identified genes related to degenerative myopia. **(A)** Enriched gene ontology (GO) terms; **(B)** Enriched pathways. BP, biological process; CC, cellular component; MF, molecular function.

**FIGURE 5:**
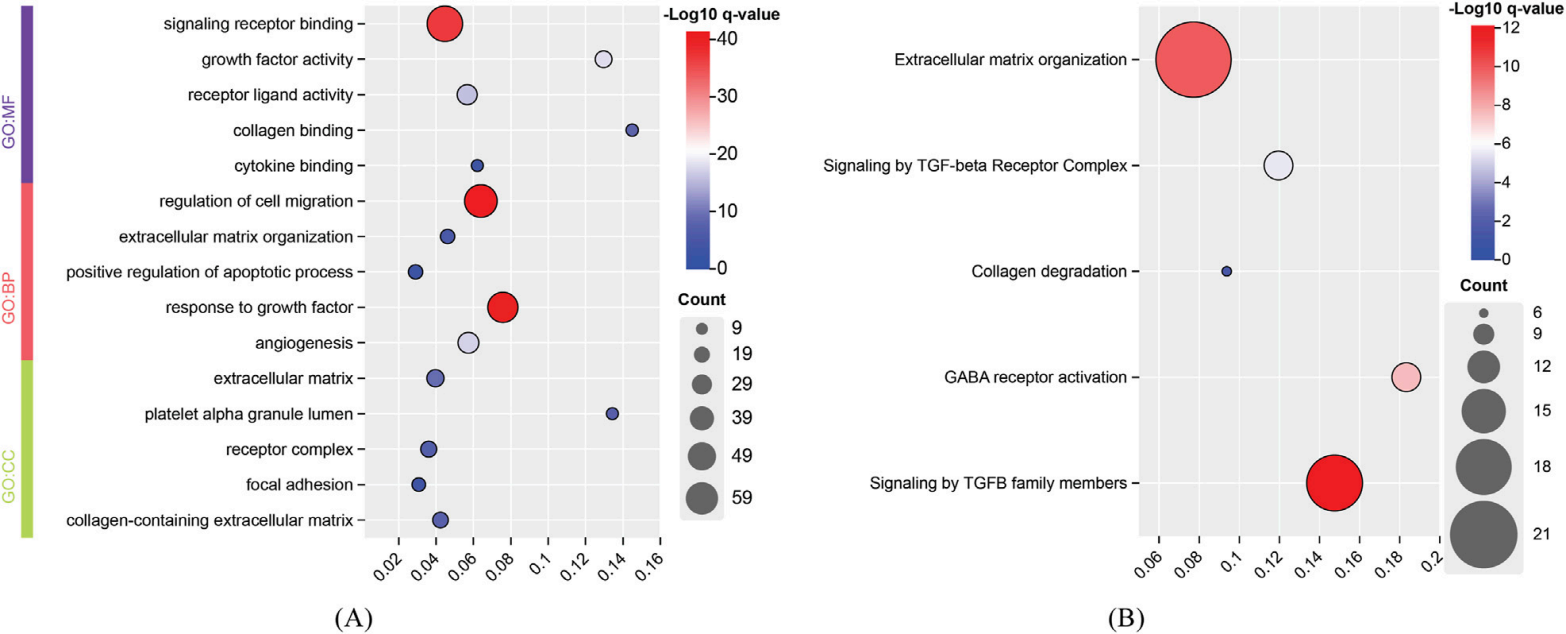
Enrichment analysis results of identified genes related to high myopia. **(A)** Enriched gene ontology (GO) terms; **(B)** Enriched pathways. BP, biological process; CC, cellular component; MF, molecular function.

For the identified genes of high and degenerative myopia, we conducted the enrichment analysis to uncover the biological meanings behind these genes. The recently proposed tool g: Profiler was employed to do this analysis (Kolberg et al., 2023), which can identify significantly enriched biological pathways and gene ontology (GO) terms for a given gene set. The enriched GO terms and pathways are listed in Figures 4, 5.

The development of degenerative myopia involves complex interactions among various signaling pathways, including those mediated by frizzled receptors, signaling receptors, and cellular responses to transforming growth factor beta (TGF-β) stimuli. Frizzled receptors, such as FZD8, are integral to the Wnt signaling pathway, which influences ocular development and may contribute to myopia progression (Murillo-Garzón et al., 2018). The development of degenerative myopia involves complex interactions among various signaling pathways, including those mediated by frizzled receptors, signaling receptors, and cellular responses to transforming growth factor beta (TGF-β) stimuli. The cellular response to TGF-β stimuli involves the activation of Smad proteins, which translocate to the nucleus to regulate gene expression. Aberrant TGF-β1 signaling activation by MAF has been implicated in pathological lens growth, a key factor in the development of high myopia (Zhu et al., 2021).

The development of high myopia is influenced by signaling receptors, cell migration, and growth factor responses, with integrins playing a key role in cell attachment and migration, particularly in ocular tissue remodeling (Meguro et al., 2020). Cell migration, particularly in the sclera, is critical for myopia progression, with EGFR signaling playing a significant role in this process (Dong et al., 2020). Growth factors like IGF1 and FGF2 regulate ocular growth, contributing to myopia, and TGF-β family members are important for early eye development and myopia pathogenesis (Shu and Lovicu, 2021). In conclusion, high myopia arises from the interplay of signaling receptors, cell migration, and growth factor responses, leading to ocular remodeling and eye elongation.

### 4.6 Medical significance of this study

The medical significance of this study includes: (1) Early screening and precision diagnosis. The newly identified genes can serve as potential biomarkers for the development of early screening tools, facilitating the differentiation between high myopia and degenerative myopia and informing timely clinical interventions. (2) Development of therapeutic targets. The identified genes and pathways (e.g., TGF-β signaling, WNT7A-PAX6 axis) offer promising avenues for targeted therapeutic development. For example: Inhibiting THBS1 may reduce retinal collagen degradation, thereby delaying degenerative progression. Modulating ADCY4 or cAMP levels may help regulate excessive axial elongation. (3) Broader methodological applications. The computational framework is extensible to other complex diseases, such as glaucoma and age-related macular degeneration, advancing the application of computational biology in ophthalmology and beyond. (4) Potential for clinical translation. The study findings provide a theoretical foundation for gene-editing approaches (e.g., CRISPR) and small-molecule drug development (e.g., targeting TGF-β receptors), potentially driving the advancement of personalized treatment strategies.

### 4.7 Limitations of this study

This study designed a computational method to identify latent biomarkers of high and degenerative myopia. Some key genes were found out. However, some limitations exist. First, the proposed method was highly relied on the PPI network. The accuracy of PPI information determined the results of this method. At present, this information is far from complete, reducing the effectiveness of the method. Second, although some identified genes can be supported by some published literature, they still need to be validated by extensively wet experiments. In future, we will continue this work to infer more reliable biomarkers.

## 5 Conclusion

In our study, GenePlexus was utilized to explore the Protein-Protein Interaction (PPI) network, with a particular emphasis on genes implicated in both high and degenerative myopia, serving as initial seed nodes. This exploration entailed the application of three distinct screening techniques, resulting in the identification of 133 proteins related to high myopia and 21 proteins linked to degenerative myopia. Importantly, genes that emerged as highly relevant to pathological myopia were either directly associated with the condition or involved in recognized pathways pertinent to myopia, such as the regulation of gap junction trafficking and electrical signaling across these cellular junctions. The validation of genetic markers identified in our research, corroborated by existing literature, underscores GenePlexus’s precision and efficacy in elucidating the biological underpinnings of myopia. This methodology not only reinforces our comprehension of myopia but also proposes an innovative approach for biomarker discovery and the examination of underlying mechanisms in other complex conditions, providing a new avenue for the exploration of disease biomarkers and the deciphering of intricate disease pathways.

## Data Availability

Publicly available datasets were analyzed in this study. This data can be found here: https://disgenet.com/.

## Supplementary material

SUPPLEMENTARY TABLE S1 Listing of the 184 genes associated with high myopia and 39 genes associated with degenerative myopia, along with annotated information sourced from DisGeNET.

SUPPLEMENTARY TABLE S2 Detailed results of screening tests, including each filtering condition, for genes associated with high myopia, presented in the corresponding sheet.

SUPPLEMENTARY TABLE S3 Detailed results of screening tests, including each filtering condition, for genes associated with degenerative myopia, presented in the corresponding sheet.

SUPPLEMENTARY TABLE S4 Intersection of genes identified by RWR and GenePlexus.
